# Risks of hypertension and thromboembolism in patients receiving bevacizumab with chemotherapy for colorectal cancer: A systematic review and meta‐analysis

**DOI:** 10.1002/cam4.6662

**Published:** 2023-12-08

**Authors:** Akshit Chitkara, Nirmaljot Kaur, Aditya Desai, Devanshi Mehta, Fnu Anamika, Srawani Sarkar, Nandini Gowda, Prabhdeep Sethi, Rajat Thawani, Emerson Y. Chen

**Affiliations:** ^1^ Internal Medicine University of California Riverside Riverside California USA; ^2^ Loma Linda University California in Internal Medicine California USA; ^3^ Internal Medicine Hackensack Meridian Ocean University Brick New Jersey USA; ^4^ Research Lab Albert Einstein College of Medicine New York New York USA; ^5^ Division of Hematology and Medical Oncology, Knight Cancer Institute Oregon Health & Sciences University Portland Oregon USA

**Keywords:** bevacizumab, colorectal neoplasms, hypertension, thromboembolism

## Abstract

**Background:**

Guidelines show that for metastatic colorectal cancer (mCRC), a combination of three‐drug regimens, fluorouracil, leucovorin, and oxaliplatin and bevacizumab (BVZ), is one of the first‐line standard therapies. BVZ is generally well tolerated; however, it is associated with infrequent, life‐threatening side effects such as severe hypertension (HTN) (5%–18%), Grade ≥3 arterial thromboembolism (ATE) (2.6%), Grade ≥3 hemorrhagic events (1.2%–4.6%), and gastrointestinal perforation (0.3%–2.4%). This meta‐analysis aims to evaluate the additive risk of BVZ‐induced severe HTN and thromboembolism when BVZ is combined with a standard chemotherapy regime in patients with mCRC.

**Methods:**

Our search was conducted from January 29, 2022, to February 22, 2022, through databases of PubMed, clinicaltrial.gov, EMBASE, Web of Science, and Cochrane Library. Data analysis from randomized controlled trials (RCTs) and clinical trials was conducted using Review Manager V.5.4, comparing BVZ‐chemotherapy to chemotherapy only, focusing on cardiovascular AE such as HTN and arterial and venous thromboembolism.

**Results:**

The analysis from 26 clinical trials and RCTs showed that the odds of HTN were about four times higher, and ATE subgroup analysis of 11 studies showed over two times higher odds of ATE in patients being treated with BVZ compared to the chemotherapy‐only group.

**Conclusion:**

BVZ, when added to the standard chemotherapy regimen for mCRC, was associated with higher odds of developing HTN and thromboembolism, specifically ATE, than the chemotherapy‐only group. Our findings are significant as they provide vital information in analyzing the risk–benefit ratio of adding BVZ to the standard chemotherapy regime in patients with mCRC, especially in patients with vascular comorbidities.

## 1 INTRODUCTION

Colorectal cancer (CRC) is the second leading cause of death related to cancer (53,200) and the fourth most frequently diagnosed cancer (147,950) in 2020, per the American Cancer Society in the United States.^7^, ^11^, ^59^

Bevacizumab (BVZ), a recombinant humanized monoclonal IgG antibody directed against the human vascular endothelial growth factor (VEGF), is commonly used in frontline to later lines of treatment in advanced or metastatic colorectal cancer (mCRC).^1^, ^6^ BVZ inhibits endothelial cell proliferation and neovascularization by inhibiting the interaction of VEGF with its receptors (Flt‐1 and KDR) situated on the surface of endothelial cells and has a long half‐life of 3–4 weeks.^2^ The connection between the vascularization of tumors and metastasis has made VEGF the most important of several growth factors to regulate angiogenesis and a target for antineoplastic agents.^8^

BVZ was first approved by the FDA (Food and Drug Administration) to be used in combination with 5‐fluorouracil (5‐FU) based chemotherapy in 2004.^3^ Subsequently, multiple trials have also shown that BVZ combined with capecitabine, irinotecan, or oxaliplatin‐based chemotherapy improved progression‐free survival and overall survival in patients with previously untreated mCRC.^1^, ^3^, ^7^, ^12^ Adding BVZ to oxaliplatin, 5‐FU, and leucovorin for patients with mCRC previously treated with chemotherapy improves survival.^13^ Patients with mCRC, on average, are likely to be exposed to BVZ for a long duration in their overall treatment course.

Treatment guidelines for advanced or mCRC show that a cytotoxic combination of fluorouracil, leucovorin, and oxaliplatin (FOLFOX) and a biologic, BVZ, is a standard first‐line choice of therapy. Other valuable first‐line chemotherapy combinations may include irinotecan such as FOLFOXIRI (folinic acid, fluorouracil, oxaliplatin, and irinotecan) or FOLFIRI (folinic acid, fluorouracil, and irinotecan) and are still often given together with BVZ.^4^, ^5^, ^60^ Subsequently, maintenance therapy with fluoropyrimidine and third‐line therapy with trifluridine/tipiracil often also include BVZ.^14^, ^15^

Side effects of BVZ are often manageable; however, it is associated with occasional, life‐threatening side effects such as severe hypertension (HTN) (5%–18%), Grade ≥3 arterial thromboembolism (ATE) (2.6%), Grade ≥3 hemorrhagic events (1.2%–4.6%), delayed wound healing, and gastrointestinal perforation (0.3%–2.4%).^1^, ^16^ The risk of ATE is higher in patients with preexisting cardiovascular risk factors or cardiovascular diseases, including myocardial infarction, chronic heart failure, or stroke. The mechanism of BVZ's increased risk of ATE is not well studied, but some studies suggest that BVZ increases the risk by promoting inflammation and atherosclerotic instability, vasoconstriction by decreasing nitrous oxide production, and platelet aggregation and adhesion to vascular endothelium by lowering endothelial cell renewal capacity.^10^

HTN of all grades has been reported in up to 36% of patients being treated with BVZ. In contrast, the reported incidence of high‐grade HTN ranges from 1.8% to 22%, with up to 1% of events being grade 4 HTN. Unmanaged HTN can lead to cardiovascular complications, subarachnoid hemorrhage, and encephalopathy. The prevailing hypothesis for the mechanism of BVZ‐induced HTN is the inhibition of VEGF‐mediated vasodilation leading to an increase in vascular tone.^9^

As we observe an upward trend in using BVZ in the first‐line treatment for patients with mCRC, it is important to analyze the side effects and potential toxicities that this drug can cause in this population. Earlier randomized controlled trials (RCTs) could not estimate the risk of thromboembolic toxicity with high precision due to low thromboembolic event rates. This meta‐analysis aims to evaluate and assess the risk of BVZ‐induced severe HTN and the odds of thromboembolic toxicity of BVZ, including subgroup analysis of FOLFOX subgroup and arterial and venous thromboembolic events with BVZ in combination with most frequently used chemotherapy regimens compared to chemotherapy without BVZ in patients with mCRC.

## 2 METHODS

This meta‐analysis of RCTs and clinical trials compared BVZ‐chemotherapy to chemotherapy only, focusing on cardiovascular AE such as HTN and arterial and venous thromboembolism. The review was registered on Prospero under registration ID CRD42021246788.

### 2.1 Search criteria

A systematic search was conducted using meta‐analyses of observational studies in epidemiology guidelines^1^ for clinical trials and RCTs of patients with colon cancer by using the following keyword/Medical Subject Headings (MeSH) terms: (("Bevacizumab"[Mesh]) AND "Colorectal Neoplasms"[Mesh]) AND (“Cardiology"[Mesh] OR "Heart Disease Risk Factors"[Mesh] OR "Cardiovascular Diseases"[Mesh]).

We included RCTs and other clinical trials from 1980 to March 2022 with BVZ added to cytotoxic chemotherapy backbone for patients with CRC if adverse event reporting included cardiovascular side effects. Both adjuvant and metastatic settings were included. We excluded all studies where patients did not receive treatment with BVZ for CRC. We excluded review articles, meta‐analyses, observational studies, or case series. Additionally, non‐English literature, animal studies, and non‐full text were excluded.

**FIGURE 1:**
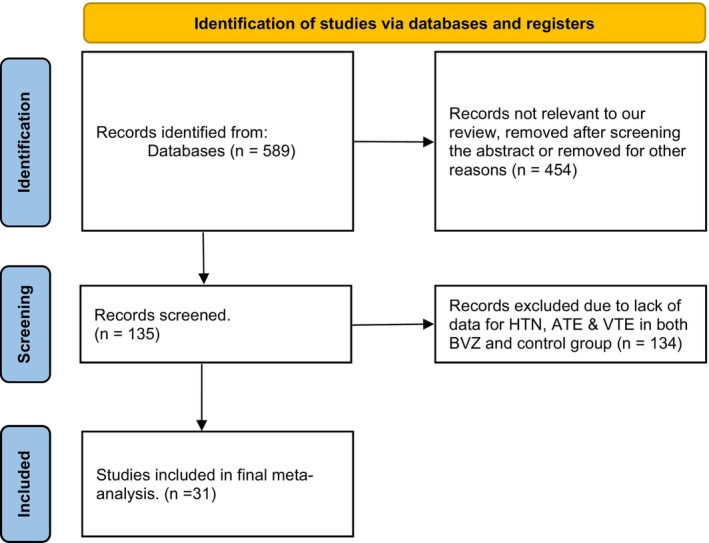
Flow chart showing study selection algorithm.

Our search was conducted from January 29, 2022, to February 22, 2022, through databases of PubMed, clinicaltrial.gov, EMBASE, Web of Science, and Cochrane Library. Abstracts were reviewed, after which full articles were checked for the availability of data on the cardiovascular side effects of BVZ in patients with CRC. Studies included in this meta‐analysis used BVZ to treat CRC and reported side effects, including cardiovascular side effects. AD and DM independently screened all studies identified and simultaneously assessed full texts to check eligibility. Any disagreements were resolved through discussion with another reviewer (AC). A flow diagram depicting the literature search and study selection process has been described in Figure 1.

### 2.2 Data extraction

We manually extracted the following variables from the included studies using prespecified data collection forms by two authors (NK and AK) with a common consensus on the disagreement of another author (AD). Quantitative data were extracted from collection forms to a Google spreadsheet. We collected the following characteristics in our study, including the first author's last name, date of publication, country, sample size, median age, sex, the dosage of BVZ, chemotherapy regimen, the incidence of grade 3 or 4 HTN, arterial and venous thromboembolism in BVZ treatment, and chemotherapy‐only control groups. Separate subgroup analyses were performed to calculate the OR of HTN in the FOLFOX subgroup, arterial and venous thromboembolism.

Before inclusion in the review, two independent reviewers (AD and DM) assessed quantitative papers selected for retrieval for methodological validity, utilizing standardized critical appraisal instruments by the Joanna Briggs Institute Meta‐Analysis of Statistics Assessment and Review Instrument. Any reviewer disagreements were resolved with discussion or with a third reviewer (AC). In case, the study has more than one outcome comparison. In that case, data from the most severe outcome in the analysis were used to minimize the study's overall selection bias.

### 2.3 Statistical analysis

Dichotomous variables were calculated using the Mantel–Haenszel formula to obtain ORs and their 95% CIs. It was used to analyze the relationship between BVZ and cardiovascular side effects (grade 3 or 4 HTN, arterial and venous thromboembolism) compared to chemotherapy alone. Regardless of heterogeneity, random‐effect models were used to estimate the combined effect and its precision to give a better conservative estimate of the ORs and 95% CI.^17^

Chi‐square and *I* ^2^ tests evaluated heterogeneities across individual studies. The *I* ^2^ statistic was utilized to analyze statistical heterogeneity. The *I* ^2^ statistic of >50% was deemed significant heterogeneity. *p*‐value < 0.05 was considered significant.

Data analysis was performed using Review Manager V.5.4 (The Nordic Cochrane Centre, The Cochrane Collaboration, Copenhagen, Denmark). To evaluate the effect of publication bias and heterogeneity by excluding outlying studies on the funnel plot, sensitivity analysis was performed. Funnel plots were visually assessed for publication bias.^18^

## 3 RESULTS

Of the 589 studies published as of March 2022, 31 RCTs and clinical trials were selected after excluding duplicates and irrelevant studies, including 17,599 patients in our study. All studies were deemed to be of moderate quality using the Cochrane risk‐of‐bias (RoB 2) tool. A total of 9609 patients received adjuvant chemotherapy plus BVZ therapy, and 7990 received adjuvant chemotherapy alone.

Patients in this study had a mean age of 65 years. The proportion of the male population was 55%. The entire analysis control group had several guideline‐directed chemotherapies, and the “FOLFOX subgroup analysis” control group focused on FOLFOX therapy alone. The baseline characteristics of all patients included in the analysis are listed in Table 1.

**TABLE 1:** Baseline characteristics of all patients included in the analysis.

PMID	First author	Year of publication	Median age (years)	BVZ + chemo (#)	Chemo only (#)	BVZ dosage (mg/kg)
17405901	Cohen, Martin H.^2^	2007	62	293	292	10
21959045	Guan, Zhong‐Zhen^3^	2011	53	141	70	5
24028813	Cunningham, David^30^	2013	76	134	136	7.5
23168366	Bennouna, Jaafar^31^	2012	63	409	409	2.5
22039086	Price, Timothy J.^32^	2011	72	315	156	No data
12506171	Kabbinavar, Fairooz^33^	2003	NA	68	35	5 & 10
19414665	Allegra, Carmen J.^34^	2009	NA	1326	1321	5
32749938	Tang, Wentao^35^	2020	NA	121	120	5
27660192	Kerr, Rachel S.^36^	2016	NA	959	968	7.5
25735317	Passardi, Alessandro^37^	2015	66	176	194	5
25481673	Cao, Ranhua^38^	2015	62	65	77	10
24687833	Schwartzberg, Lee S.^39^	2014	61	143	142	5
23168362	Gramont, Aimery D.^40^	2012	58	2300	1151	5 & 7.5
22294255	Dotan, Efrat^41^	2012	59	12	11	7.5
17442997	Giantonio, Bruce J.^42^	2016	60.8	529	291	10
15908660	Hurwitz, Herbert I.^43^	2016	59.7	110	100	5
15738537	Kabbinavar, Fairooz F.^44^	2005	71.3	104	105	5
15175435	Hurwitz, Herbert^45^	2004	59.5	402	411	5
20798560	Stathopoulos, George P.^46^	2010	67	114	108	7.5
20516443	Tebbutt, Niall C.^47^	2010	67	315	156	7.5
28258825	Kapelakis, Ioannis^48^	2017	64.3	38	16	7.5
18421054	Saltz, Leonard B.^49^	2008	60	699	701	5 & 7.5
18640933	Hochster, Howard S.^50^	2008	61.5	213	147	5 & 7.5
19382200	Jackson, Nadine A.^51^	2009	65	115	274	5 & 7.5
19940012	Sharma, Sunil^52^	2010	62	16	14	1, 2 & 5
21189384	Kemeny, Nancy E.^53^	2011	NA	35	38	5
Madajewicz	Madajewicz, Stefan^54^	2012	63	36	48	5
22219013	Stintzing, Sebastian^55^	2012	65	46	50	5
22965961	Schmoll, Hans‐Joachim^56^	2012	60	713	709	5
23299530	Cunningham, David^57^	2013	NA	66	144	10
25088940	Heinemann, Volker^58^	2014	65	295	297	5

Abbreviation: NA, not available.

**FIGURE 2:**
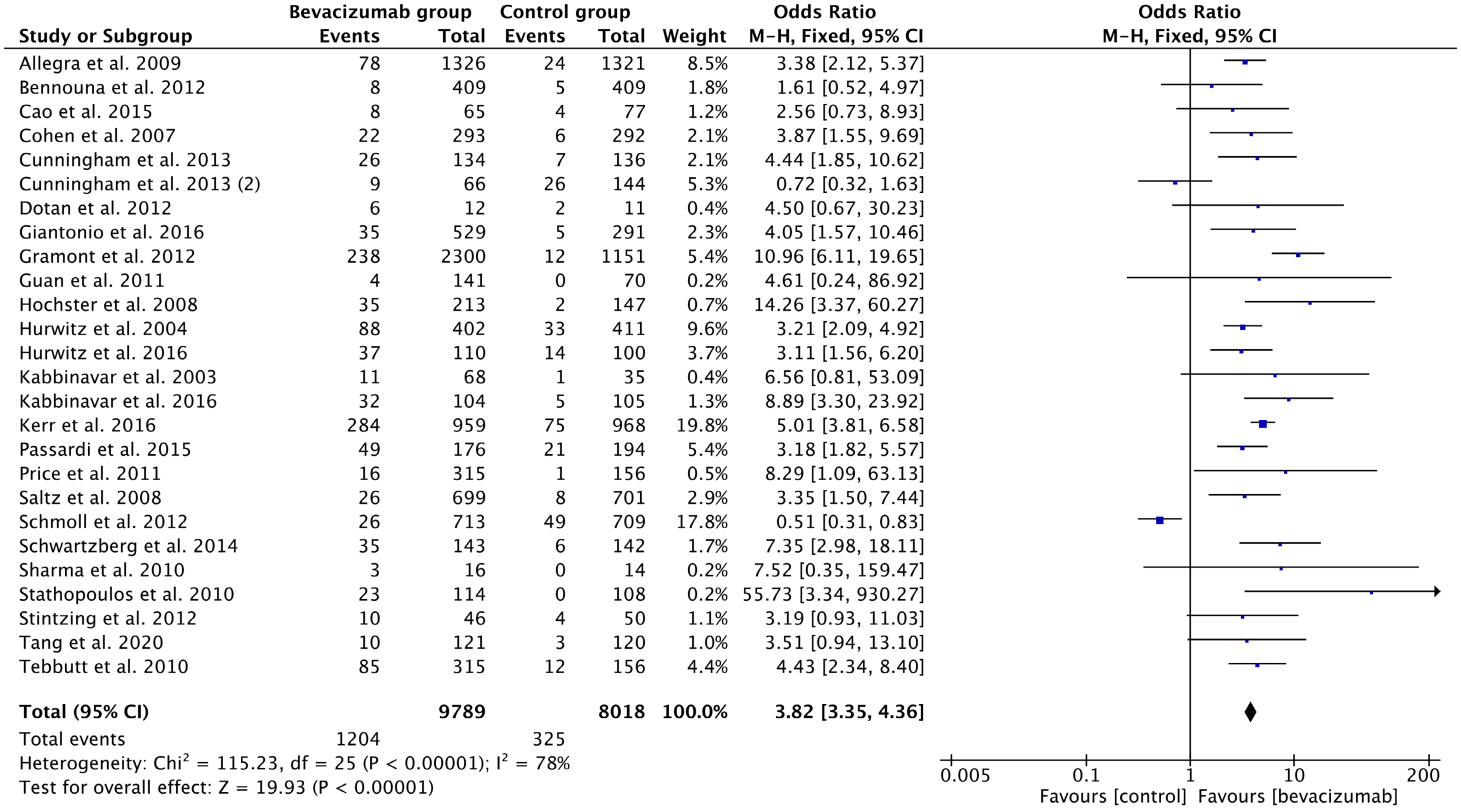
Forest plot showing odds of HTN in the BVZ group versus chemo‐only group. BVZ, bevacizumab; HTN, hypertension.

The analysis (Figure 2) pooled from 26 clinical trials, and RCTs showed that the odds of HTN were about four times higher (OR 3.82, 95% CI 3.35–4.36, *p‐*value < 0.00001, *I* ^2^ = 78%) in 9789 patients treated with BVZ (12.3%) than the 8018 patients in the control group (4%). Of the 26 studies included in this analysis, 24 suggest higher odds of HTN, while 2 suggested lesser odds of HTN in the BVZ group than in the chemotherapy‐only group.

**FIGURE 3:**
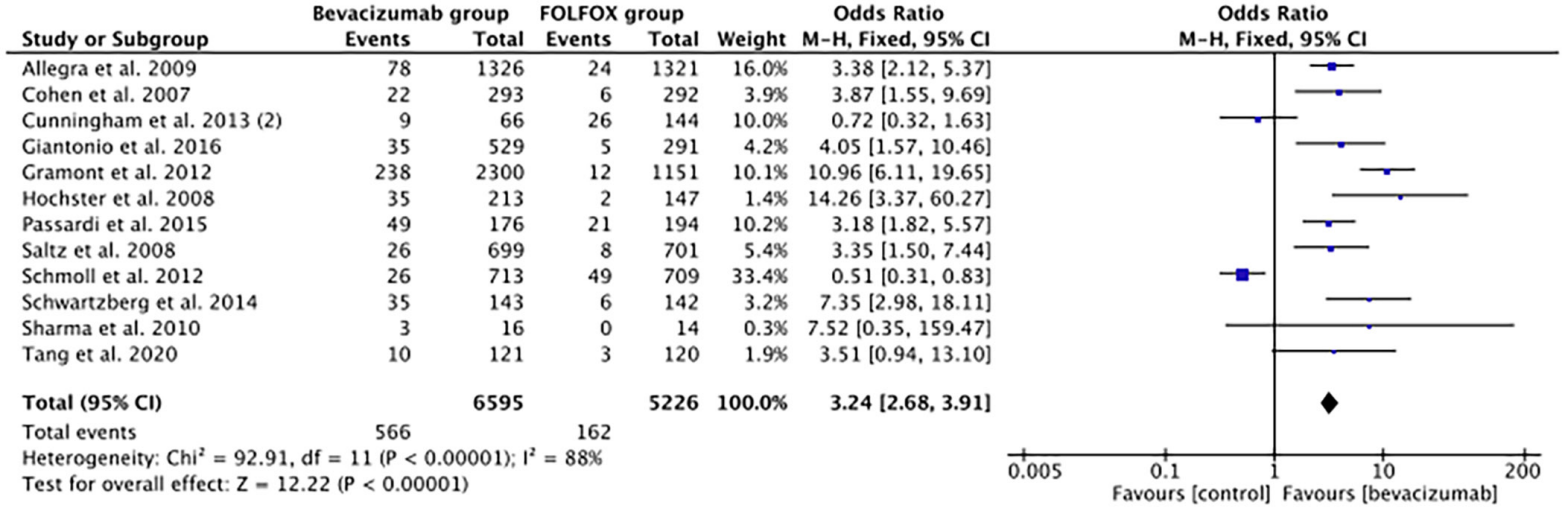
Forest plot showing odds of HTN in the BVZ group versus FOLFOX‐only subgroup. BVZ, bevacizumab; FOLFOX, fluorouracil, leucovorin, and oxaliplatin; HTN, hypertension.

In a subgroup analysis (Figure 3) of 12 studies with FOLFOX chemotherapy backbone, comparing BVZ + FOLFOX to FOLFOX, the odds of severe HTN were more than five times higher (OR 5.24, 95% CI 4.06–6.77, *p*‐value < 0.00001, *I* ^2^ = 58%) in the 6596 patients of the BVZ group (8.6%) than 5226 patients in the FOLFOX group (3.1%).

**FIGURE 4:**
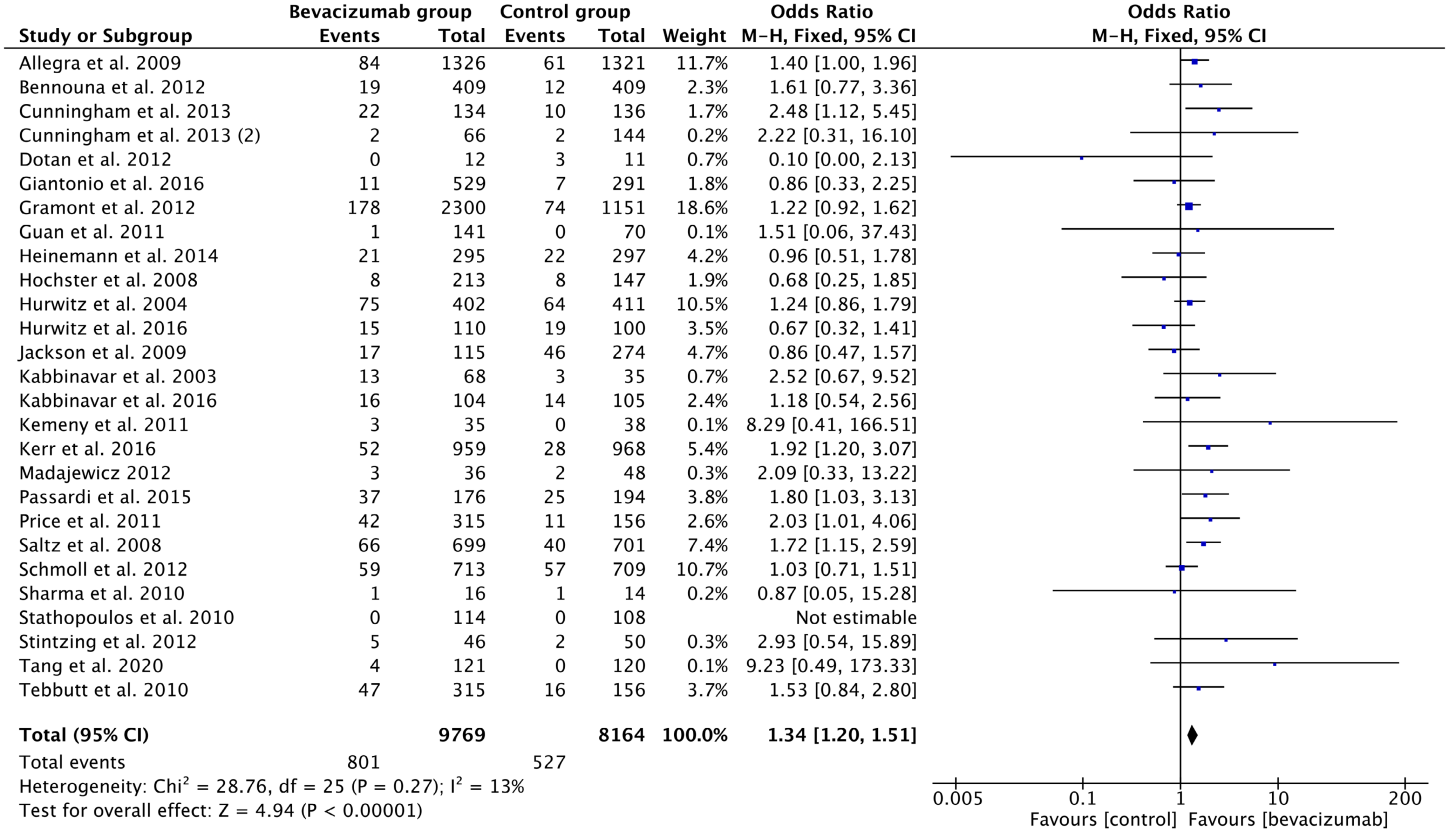
Forest plot showing odds of thromboembolism in the BVZ group versus chemo‐only group. BVZ, bevacizumab.

A meta‐analysis (Figure 4) of 29 clinical trials and RCTs suggests that the odds of thromboembolism in 9769 patients managed with BVZ (8.2%) was about 34% higher (OR 1.34, 95% CI 1.20–1.51, *p*‐value < 0.00001, *I* ^2^ = 13%) than the 8164 patients in the control group (6.45%).

**FIGURE 5:**
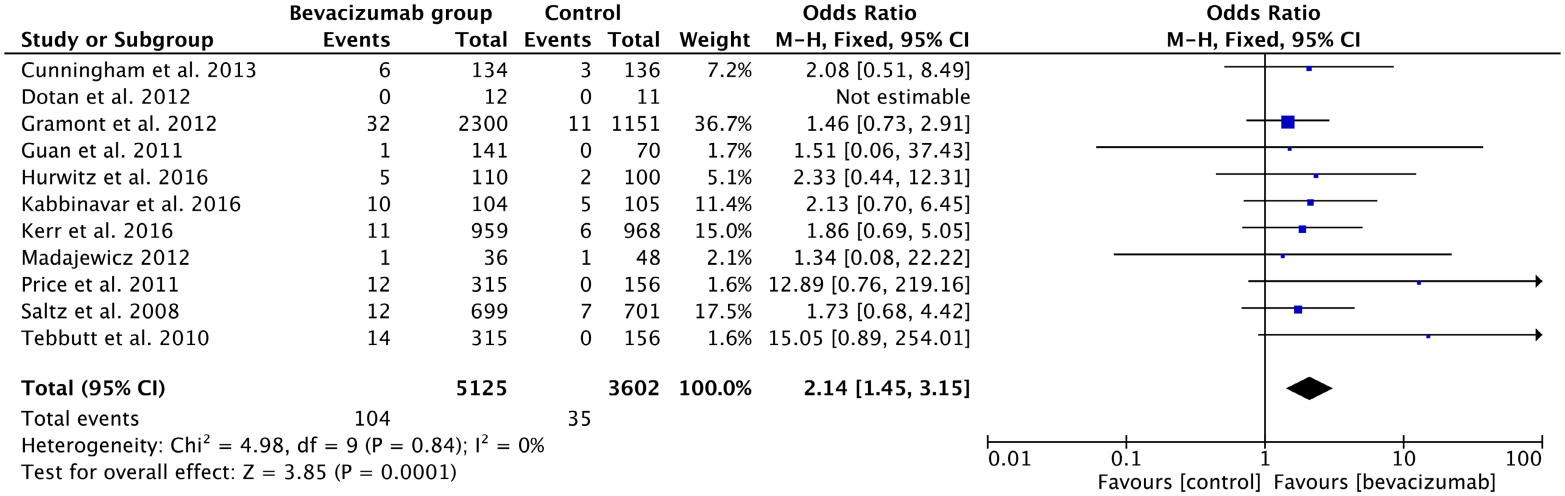
Forest plot showing odds of ATE in the BVZ group versus chemo‐only group. ATE, arterial thromboembolism; BVZ, bevacizumab.

Further, we did a subgroup analysis to study the odds of ATE and VTE in the BVZ+ chemotherapy group versus chemotherapy only. In the ATE subgroup analysis (Figure 5), we included 11 studies comprising 5125 patients in the BVZ + Chemotherapy group and 3602 in the control group. Our analysis showed over two times higher odds of ATE (OR 2.14, 95% CI 1.45–3.15, *p*‐value < 0.00001, *I* ^2^ = 0%) in patients being treated with BVZ (2%) as compared to the chemotherapy‐only group (1%).

**FIGURE 6:**
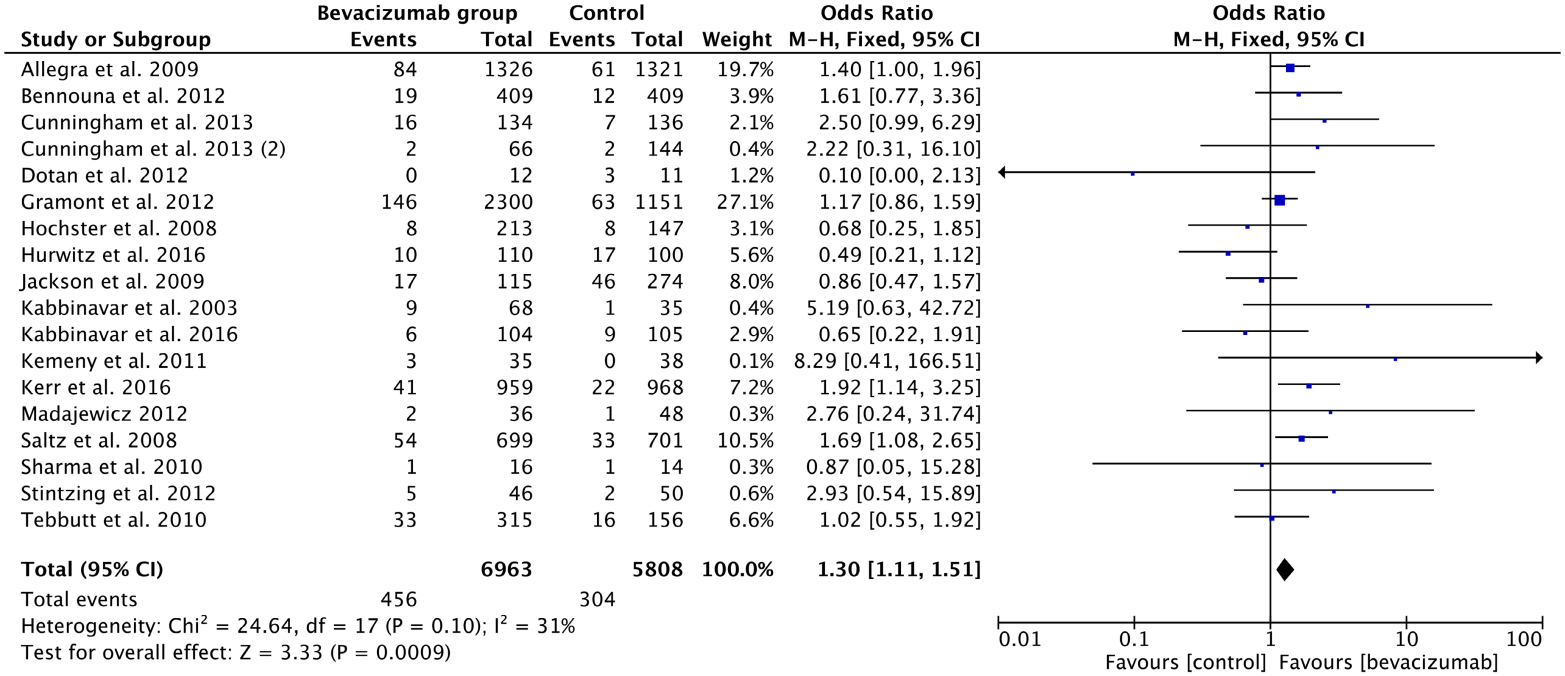
Forest plot showing odds of VTE in the BVZ group versus chemo‐only group. BVZ, bevacizumab.

The third subgroup analysis (Figure 6) to study the odds of VTE included 18 studies comprising 6963 patients in the BVZ + Chemotherapy group and 5108 in the chemo‐only control group. We calculated that the odds of VTE in patients being treated with BVZ + Chemotherapy (6.55%) were around 1.3 times higher (OR 1.30, 95% CI 1.11–1.51, *p*‐value < 0.0009, *I* ^2^ = 31%) than the chemotherapy‐only group (5.2%).

**FIGURE 7:**
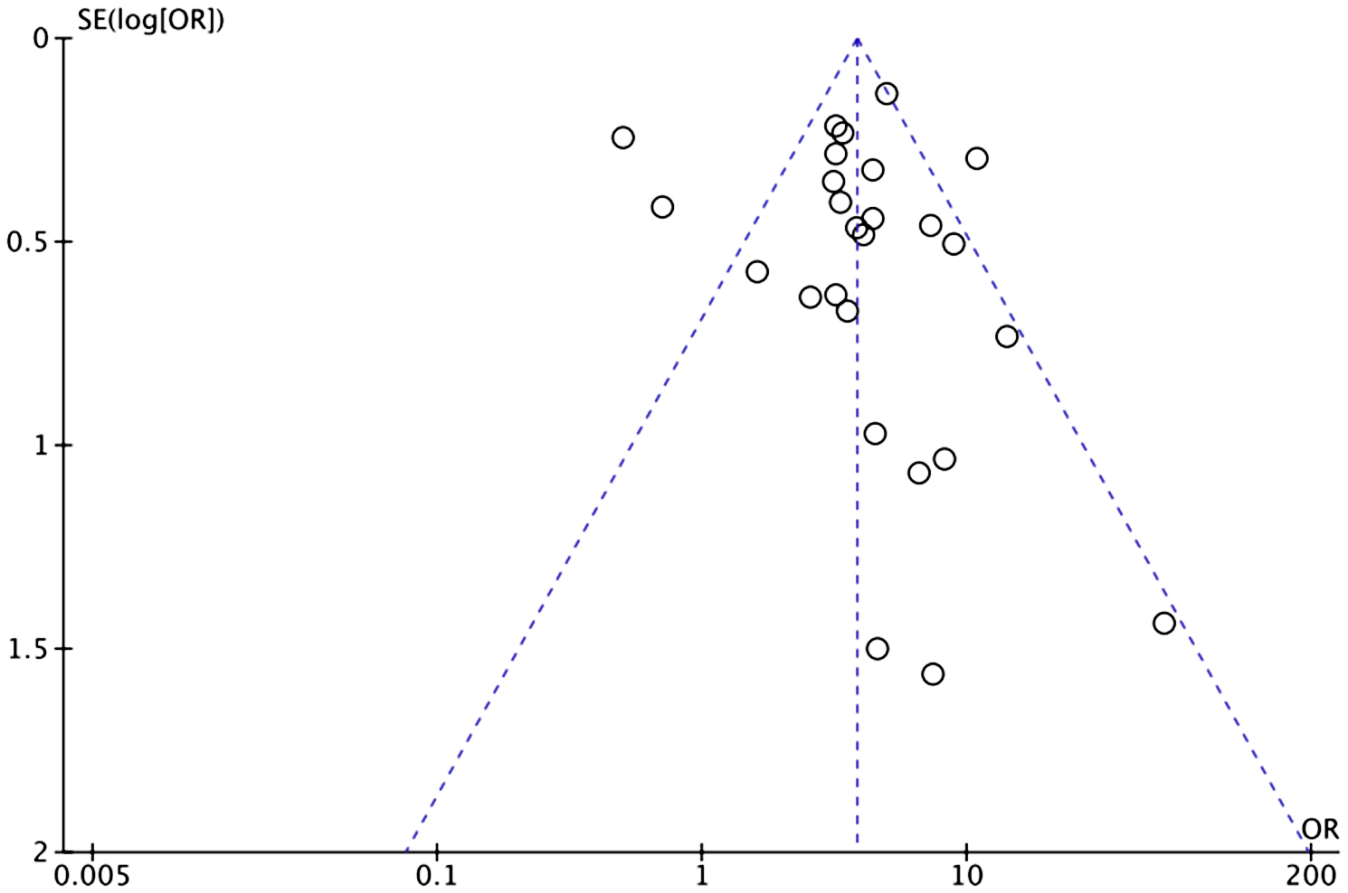
Funnel plot showing OR of HTN in the BVZ group versus chemo‐only group with asymmetrical distribution. BVZ, bevacizumab; HTN, hypertension.

**FIGURE 8:**
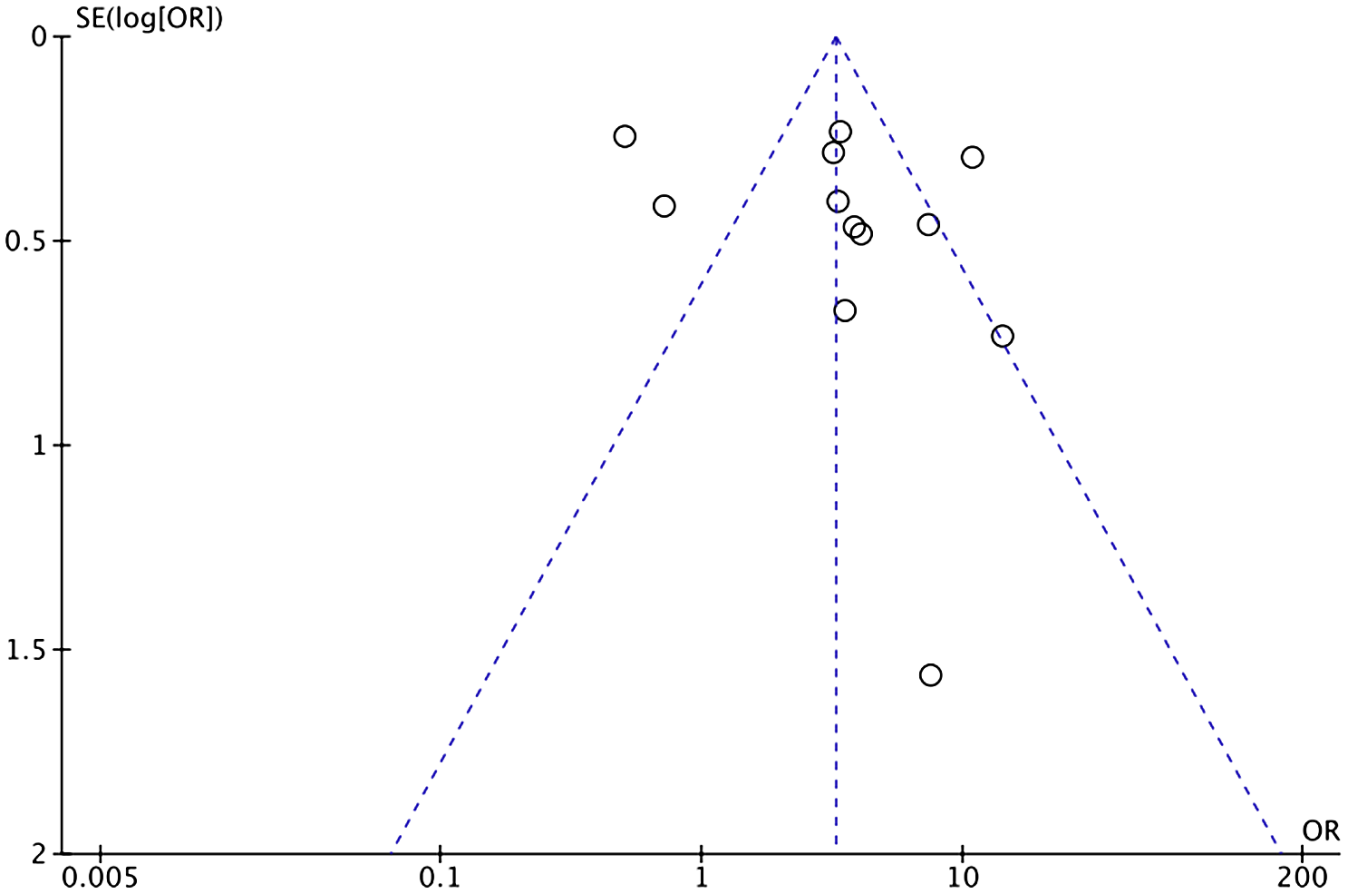
Funnel plot showing OR of HTN in the BVZ group versus FOLFOX‐only group with asymmetrical distribution. BVZ, bevacizumab; FOLFOX, fluorouracil, leucovorin, and oxaliplatin; HTN, hypertension.

**FIGURE 9:**
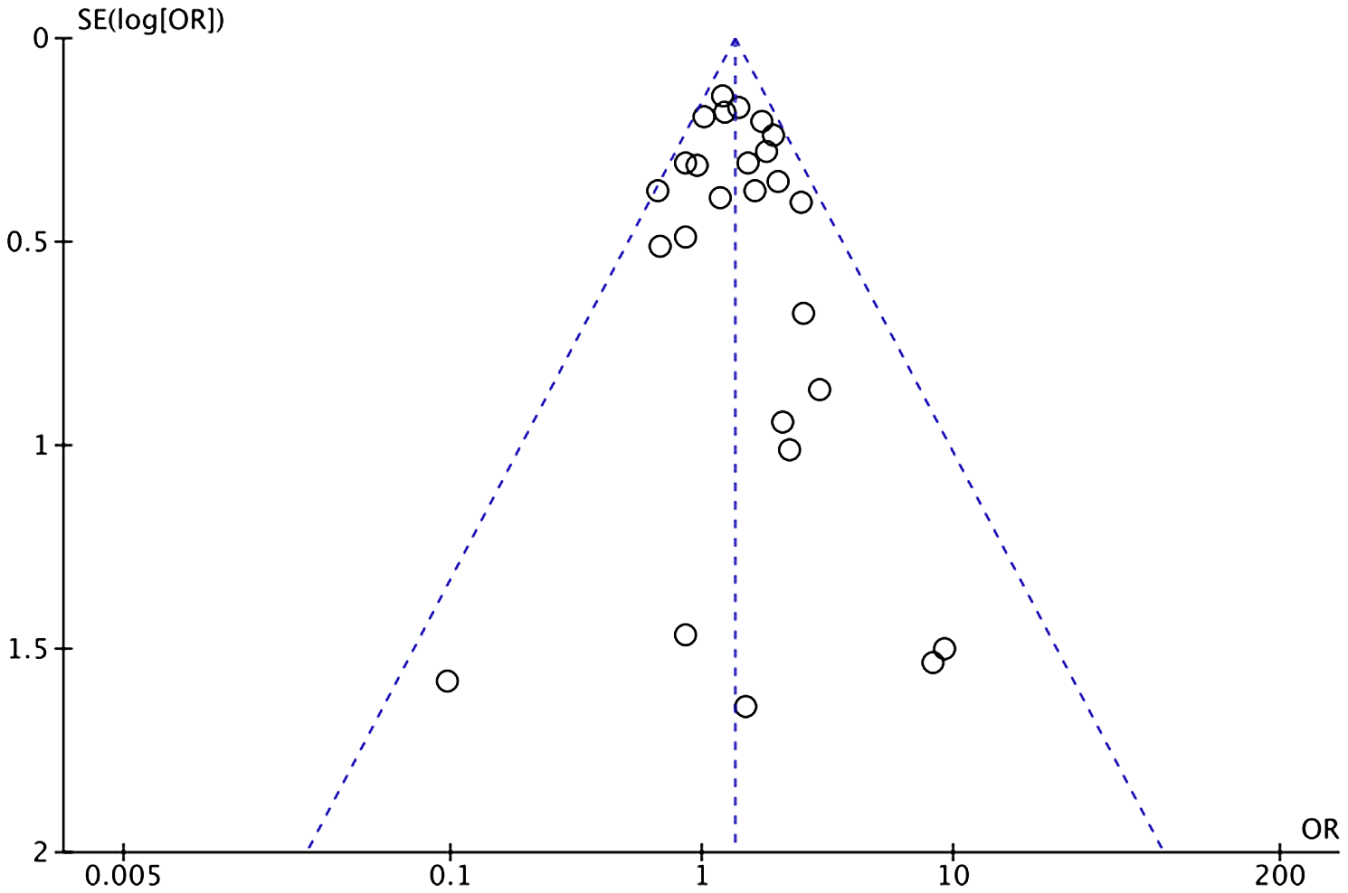
Funnel plot showing OR of thromboembolism in the BVZ group versus chemo‐only group with symmetrical distribution. BVZ, bevacizumab.

**FIGURE 10:**
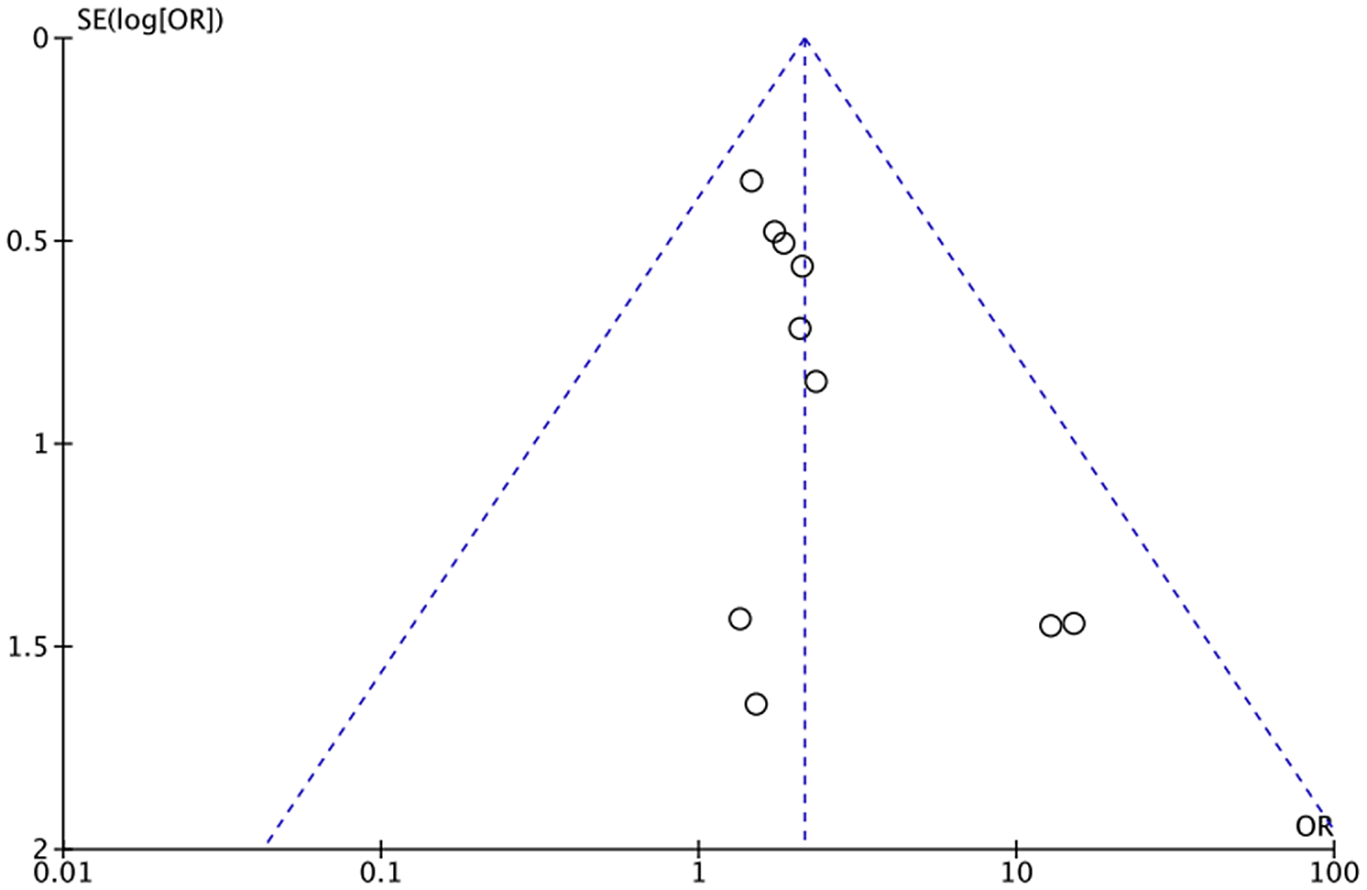
Funnel plot showing OR of ATE in the BVZ group versus chemo‐only group with asymmetrical distribution. ATE, arterial thromboembolism; BVZ, bevacizumab.

**FIGURE 11:**
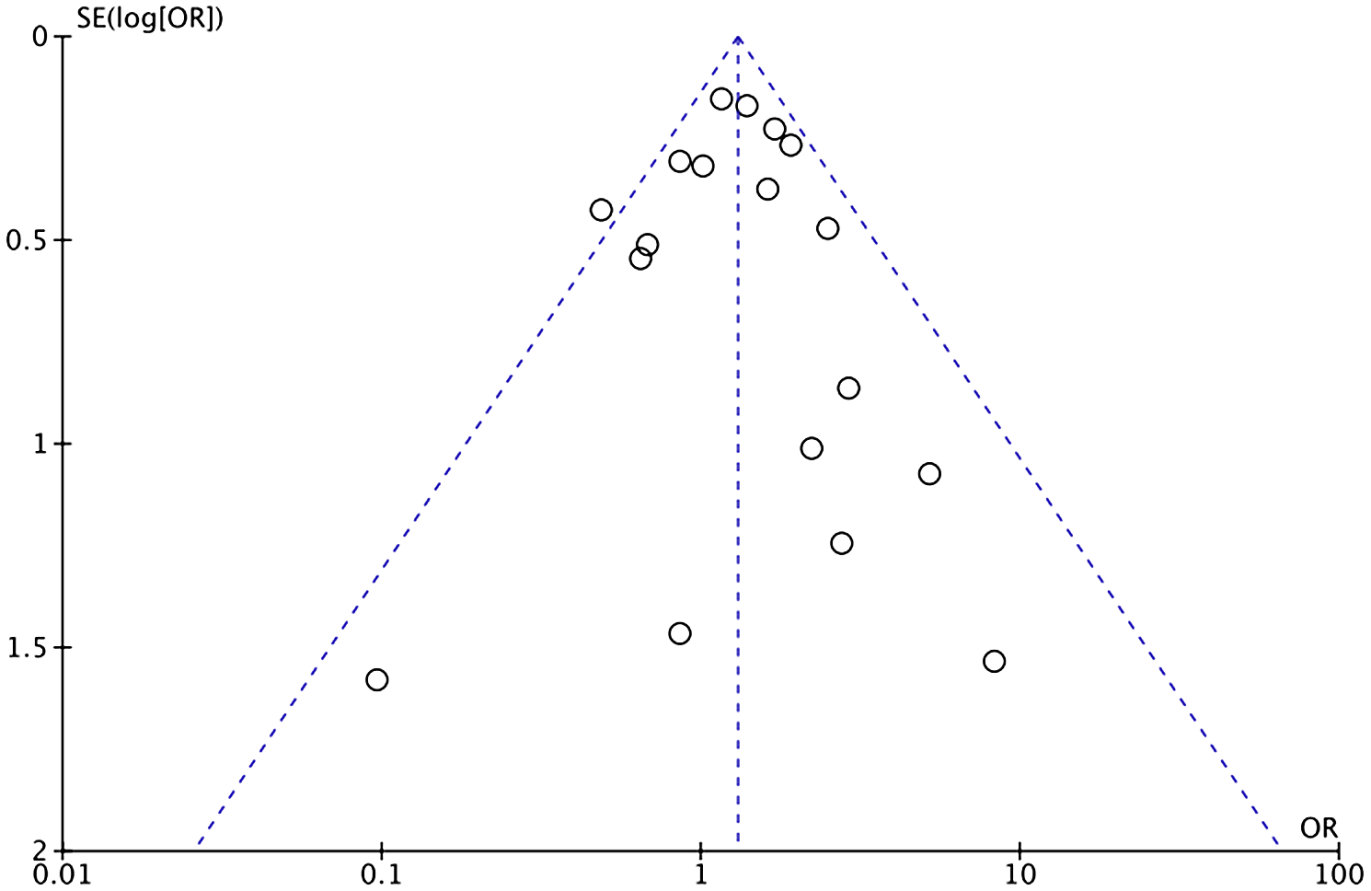
Funnel plot showing OR of VTE in the BVZ group versus chemo‐only group with asymmetrical distribution. BVZ, bevacizumab.

Heterogeneity among study results can be attributed to all stages of CRC being considered and comorbidities such as HTN, diabetes, ischemic heart disease, and previous cerebrovascular accident/TIA. Respective funnel plots have been added as supplementary data to assess the symmetry and potential risk of bias (Figures 7, 8, 9, 10, 11).

## 4 DISCUSSION

BVZ has been used to treat more than 1.56 million patients with mCRC worldwide.^19^ Previous RCTs have shown that BVZ, in combination with a standard cytotoxic regime, is safe to administer and without any significant risk of added toxicities. Cardiovascular causes are the number one cause of mortality across the globe, and we need to prevent severe HTN and ATE‐associated mortality with BVZ. Our meta‐analysis is an up‐to‐date study of cardiovascular side effects of BVZ with the largest cohort of patients in the treatment and control groups, increasing the power of the analysis. We analyzed patients across multiple subgroups, taking into account potential confounding factors, to obtain a more refined odds ratio for the given subgroup of patients.

We demonstrated that 12.3% of patients develop HTN, 8.2% develop thromboembolism, and 2% develop ATE with BVZ when added to the standard chemotherapy regimen for mCRC. The critical findings in our systematic review and meta‐analysis are as follows. First, BVZ was associated with about four times higher risk of developing HTN than the chemotherapy‐only group. The FOLFOX subgroup analysis, based on the regime used, demonstrated about five times higher odds of developing HTN (Grade 3 or more) in the BVZ + FOLFOX group compared with the FOLFOX‐only group. Our findings are supported by a previous meta‐analysis that reported increased odds of HTN up to five times with BVZ used for CRC and other indications.^20^, ^21^, ^22^ The proposed mechanism for BVZ‐induced HTN, a well‐known side‐effect of the drug, is increased vascular resistance due to the inhibition of vasodilation mediated by vascular endothelial growth factor. However, it has yet to be established as the sole factor for this effect.^9^

Our study gives the precise odds of developing grade 3–4 HTN with BVZ, which is essential because, in the literature, a few cases of hypertensive crisis with encephalopathy and subarachnoid hemorrhage have been reported.^24^, ^25^, ^26^ The current AHA guidelines recommend initiation or up‐titration of treatment for secondary HTN due to angiogenesis inhibition by BVZ.^23^ An earlier study has shown that temporary discontinuation of BVZ can bring the blood pressure level to pretreatment levels,^28^ and such a temporary hold is recommended in cases of refractory HTN on the maximum tolerated antihypertensive regime and hypertensive emergency.^27^ Prevention and management of BVZ‐specific HTN is an important aspect of best supportive care during the active treatment phase for mCRC.

Second, the odds of thromboembolism in patients managed with BVZ were over 30% higher than in the control group. Our meta‐analysis demonstrates a significant arterial thromboembolic risk of BVZ when added to the advanced CRC treatment standard regime. We found that the odds of getting ATE with BVZ were over two times higher than the chemotherapy‐only control groups. These results affirm the manufacturer's ATE claims and are an area of further research.^27^ Previously, studies have tried to evaluate the prophylactic role of aspirin in preventing ATE in patients receiving BVZ but could not conclude.^28^ Establishing guidelines to anti‐coagulate patients getting BVZ should be studied. Preventing ATE is crucial in improving the morbidity and mortality associated with BVZ treatment.

Our study had several limitations: Firstly, RCTs keep strict inclusion and exclusion criteria. Only patients with appropriate major organ functions are included in these trials; therefore, actual patients may not be represented by the results of this meta‐analysis, and our results may not be applicable to the general population in daily practice. Second, although all the studies included RCT, there was a significant difference in the timing and the primary endpoint. The follow‐up time is different from study to study, and there might be unreported events of HTN and thromboembolism after the study was concluded. Third, the studies included in our analysis were heterogeneous regarding patient characteristics across the studies. Fourth, potential differences among the trials may exist as HTN events are collected for each individual trial, such as various international institutions and administration schedules of BVZ increasing the clinical heterogeneity and difficulty in interpreting the analysis results. Finally, the treatment designs varied, and the individual patient data were not focused on in the study. Compared with individual patient data analyses, meta‐analyses based on published data tend to overestimate the treatment effects.^29^ Future prospective studies reporting individual subgroup AE data with recurrent, advanced, or mCRC in adjuvant and metastatic settings will help focus on specific subgroups and formulate customized treatment guidelines.

There is a significant risk of publication bias based on the funnel plot obtained regarding the review process. Secondly, the incidence of BVZ‐associated HTN, because of the definition of the Common Terminology Criteria for Adverse Events (CTCAE), may have been underestimated. Per the CTCAE, blood pressure greater than 150/100 mmHg or diastolic pressure increased by more than 20 mmHg is considered hypertensive. In some studies, these strict criteria would have reduced the number of hypertensive patients noted compared with the clinical criteria for the diagnosis of HTN (140/90 mmHg).

CRC is a leading cause of cancer‐related death in the United States. Most patients with mCRC are likely to receive BVZ with adjuvant chemotherapy. Our findings are significant as they provide vital information in assessing the risk–benefit ratio of adding BVZ to the standard chemotherapy in patients with mCRC, especially in patients with vascular comorbidities. Cardiovascular AEs are crucial in determining all‐cause mortality in patients with mCRC.

The magnitude of severe HTN, thromboembolic events, and cardiovascular morbidity demonstrated from our meta‐analysis informs oncology clinicians to regularly inform patients and monitor patients with CRC receiving chemotherapy with BVZ. Prevention and management of BVZ‐specific HTN and the ATE episodes in the first place can be essential in mitigating the morbidity and mortality associated with BVZ treatment and is an area of future research in this patient population. Establishing evidence‐based guidelines for blood pressure management and anti‐thrombotic medications will be important for both clinicians and researchers in the future.

## AUTHOR CONTRIBUTIONS

**Akshit Chitkara:** Conceptualization (lead); data curation (supporting); formal analysis (equal); methodology (equal); project administration (lead); supervision (lead); writing – original draft (supporting); writing – review and editing (equal). **Nirmaljot Kaur:** Data curation (supporting); formal analysis (equal); methodology (equal); software (equal); writing – original draft (supporting). **Aditya Desai:** Data curation (equal); writing – original draft (equal). **Devanshi Mehta:** Data curation (equal); writing – original draft (equal). **Fnu Anamika:** Project administration (equal); writing – review and editing (equal). **Srawani Sarkar:** Conceptualization (supporting); methodology (equal); software (equal). **Nandini Gowda:** Supervision (equal); writing – review and editing (equal). **Prabhdeep Sethi:** Resources (equal); writing – review and editing (equal). **Rajat Thawani:** Project administration (equal); supervision (equal). **Emerson Y. Chen:** Supervision (equal); validation (equal); visualization (equal); writing – review and editing (equal).

## FUNDING INFORMATION

None.

## CONFLICT OF INTEREST STATEMENT

I, Akshit Chitkara, on behalf of all coauthors, declare that I have no financial or personal conflicts of interest related to this work or its publication. My involvement in this work was solely for academic or professional purposes. I did not receive any financial or other benefits that could be perceived as a conflict of interest. Furthermore, I affirm that I have disclosed any financial or personal relationships with individuals or organizations that could have influenced my work on this project. If any potential conflicts of interest arise in the future, I will promptly disclose them and take appropriate actions to mitigate any potential influence they may have on the work.

## Data Availability

NA.
